# Urinary α_1_-Antichymotrypsin: A Biomarker of Prion Infection

**DOI:** 10.1371/journal.pone.0003870

**Published:** 2008-12-05

**Authors:** Gino Miele, Harald Seeger, Denis Marino, Ralf Eberhard, Mathias Heikenwalder, Katharina Stoeck, Max Basagni, Richard Knight, Alison Green, Francesca Chianini, Rudolf P. Wüthrich, Christoph Hock, Inga Zerr, Adriano Aguzzi

**Affiliations:** 1 Department of Pathology, UniversitätsSpital Zürich, Institute of Neuropathology, Zürich, Switzerland; 2 Prion Diagnostica Srl, Rho, Italy; 3 The National Creutzfeldt-Jakob Disease Surveillance Unit, Western General Hospital, Edinburgh, United Kingdom; 4 Moredun Research Institute, Pentlands Science Park, Edinburgh, United Kingdom; 5 UniversitätsSpital Zürich, Clinic for Nephrology, Zürich, Switzerland; 6 Division of Psychiatry Research, University of Zürich, Zürich, Switzerland; 7 National TSE Reference Center, Department of Neurology, Medical Faculty, Georg-August University, Göttingen, Germany; Imperial College London, United Kingdom

## Abstract

The occurrence of blood-borne prion transmission incidents calls for identification of potential prion carriers. However, current methods for intravital diagnosis of prion disease rely on invasive tissue biopsies and are unsuitable for large-scale screening. Sensitive biomarkers may help meeting this need. Here we scanned the genome for transcripts elevated upon prion infection and encoding secreted proteins. We found that α_1_-antichymotrypsin (α_1_-ACT) was highly upregulated in brains of scrapie-infected mice. Furthermore, α_1_-ACT levels were dramatically increased in urine of patients suffering from sporadic Creutzfeldt-Jakob disease, and increased progressively throughout the disease. Increased α_1_-ACT excretion was also found in cases of natural prion disease of animals. Therefore measurement of urinary α_1_-ACT levels may be useful for monitoring the efficacy of therapeutic regimens for prion disease, and possibly also for deferring blood and organ donors that may be at risk of transmitting prion infections.

## Introduction

The prion [1] is the infectious agent causing transmissible spongiform encephalopathies, which include sporadic (sCJD) and variant Creutzfeldt-Jakob disease (vCJD) in humans, scrapie in sheep, bovine spongiform encephalopathy (BSE) in cattle, and chronic wasting disease (CWD) in cervids [2]. Although prions may replicate in extraneural tissues [3], [4], cellular damage is essentially limited to the central nervous system (CNS). The molecular mechanisms underlying prion replication and subsequent neural damage are not entirely understood. No effective therapeutic strategies are available.

An essential component of the prion is PrP^Sc^, an abnormally folded, aggregated isoform of the host protein PrP^C^ [5]. To date, all validated laboratory assays for prion diseases rely on the immunochemical detection of PrP^Sc^. These methods are highly specific, but suffer from limited sensitivity as one infectious prion may be equivalent to <10^2^ aggregated PrP^Sc^ molecules [6] – which is much lower than the threshold of detection of most immunoassays. Additionally, the presence of excess PrP^C^ in complex biological fluids may confound immunochemical detection, even if biophysical detection methods are employed. Consequently, while positive detection of PrP^Sc^ suffices to establish a firm diagnosis of prion infection, its absence by no means rules out the presence of prion infectivity.

The hundreds of iatrogenic transmissions through organ extracts [7], contaminated surgical instruments [8], and probably through blood transfusions [9], [10] have tragically highlighted the current inability of diagnosing presymptomatic prion infections. While there has been recent progress in detecting low amounts of PrP^Sc^ in blood of experimentally inoculated hamsters by protein misfolding cyclic amplification [11], it is unknown whether this technology possesses adequate sensitivity and throughput for prion detection in human blood.

The use of surrogate biomarkers represents a diagnostic strategy fundamentally different to those delineated above. Since they typically identify secondary host reactions, surrogate biomarkers of prion infection cannot aspire at matching the specificity of PrP^Sc^ detection. On the other hand, surrogate biomarkers may be useful for identifying subjects at risk, and specifying acceptance or deferral of blood donations. In such cases high sensitivity (i.e. the identification of all suspect individuals) is more important than absolute diagnostic specificity, as the latter can be supplied by confirmatory assays.

Surrogate biomarkers may represent proteins that are differentially expressed or represented in body fluids of prion-affected individuals. S-100, neuron-specific enolase, and 14-3-3 protein have been reported to be elevated in cerebrospinal fluid (CSF) of sCJD patients [12], [13], [14]. These proteins may represent consequences of CNS damage and neuronal death. The cysteine proteinase inhibitor cystatin C was also reported to be elevated in CSF of sCJD patients [15], [16].

In an effort to characterize the transcriptome of prion-infected murine tissues, we have searched for transcripts which (1) are profoundly upregulated and (2) whose predicted gene products contain secretory leader peptides. One transcript fulfilling these criteria is serpin-*a3n*, the murine homolog of human alpha-1-antichymotrypsin (α_1_-ACT), a serine proteinase inhibitor which has been previously implicated in the pathogenesis of Alzheimer's disease [17], [18] and which has been reported to be a candidate plasma and CSF biomarker of Alzheimer's disease (AD) [19], [20], [21], [22]. In the present study, we sought to investigate the potential usefulness of α_1_-ACT, and to compare it to the recently proposed cystatin C, as candidate biomarker for prion diagnostics in body fluids.

## Results

### Transcriptional microarray analysis of whole brains from prion-infected and mock-infected mice

We have performed a genomie-wide interrogation of the brain transcriptome of C57Bl/6 mice at 145 days post inoculation (dpi) intraperitoneally (i.p.) with scrapie prions (RML strain, passage 5). In this model of murine scrapie, the incubation period between the date of inoculation and the onset of terminal clinical signs of scrapie is approximately 200 dpi.

The extracerebral inoculation route and the 145 dpi time point were chosen in order to minimize any severe histopathological changes to the brain, thereby lowering the occurrence of unspecific transcriptional changes. This goal was essentially achieved, since the vast majority of transcripts were quantitatively unaffected in prion-infected vs. mock-infected brains. When applying stringent filter settings, we identified a total of only 77 transcripts which appeared up- or down-regulated with a confidence limit of >90% and Student's *t* test *p* values of <0.05 (**Table S1** online).

The majority of these changes in expression were modest (−2.04 to +3.41 fold). Some transcripts, including glial fibrillary acidic protein, complement components, and beta-2-microglobulin, had been previously identified as being differentially expressed following prion infection [23], [24], [25], [26], [27]. Serpin*-a3n* was identified as a very highly overexpressed transcript in brains of prion-infected mice before the onset of clinical signs. The latter observation was particularly intriguing in view of the fact that Serpin-*a3n* encodes a secreted protein which is detectable in a variety of body fluids. This suggested that Serpin-*a3n* may represent a candidate biomarker for preclinical diagnosis of prion infections in cerebrospinal fluid (CSF) or serum.

### Serpin-*a3n*/α_1_-ACT transcription in brain during prion pathogenesis

**Figure 1:**
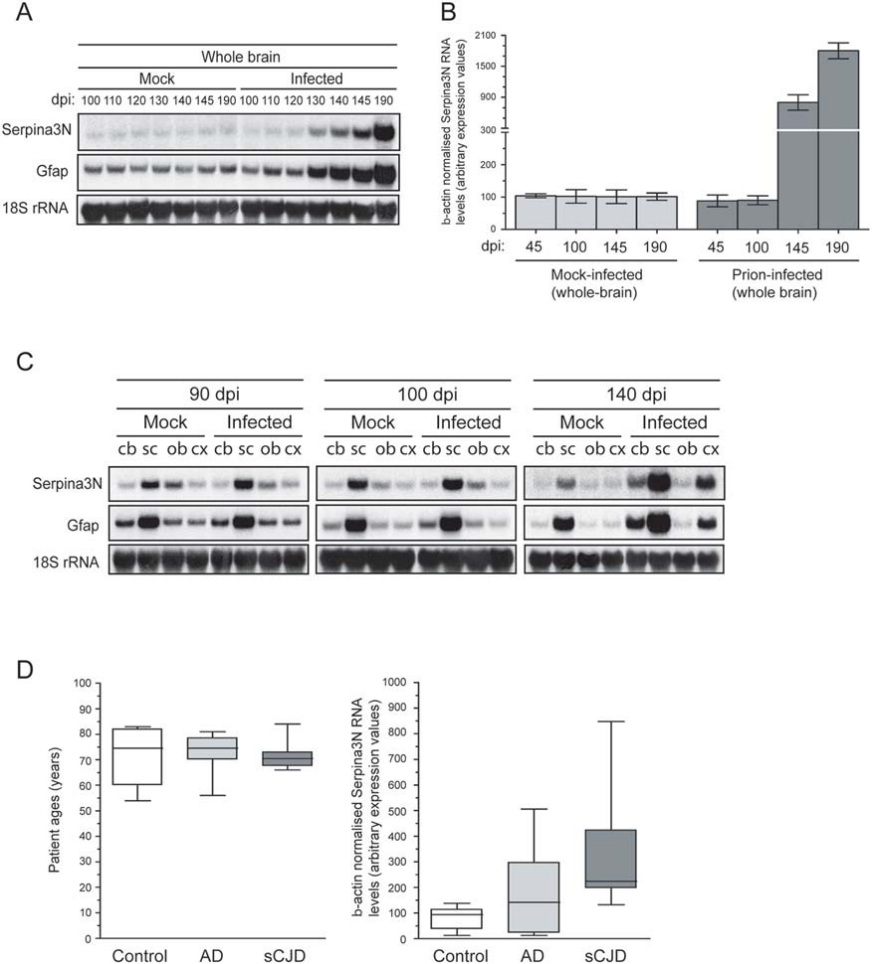
Serpin-*a3n*/α_1_-ACT transcripts in brain during prion pathogenesis. (A) Northern hybridization analysis of Serpin-*a3n* RNA expression levels in whole brains of mice throughout pathogenesis following intraperitoneal inoculation with either mock inoculum or inoculum containing 6 log LD50 RML5 mouse prions (dpi; days post-inoculation). For comparison, expression levels of Glial fibrillary acidic protein (Gfap) RNA are also shown. Expression levels were normalized to 18S rRNA levels. Differential expression of Serpin-*a3n* transcripts is evident between 120 dpi and 130 dpi in whole brain. For comparison, increased RNA levels of Gfap, traditionally thought of as one of the largest gene expression changes during prion pathogenesis, is evident between 120 dpi and 130 dpi and rises to approximately 8-fold by 190 dpi. (B) DNAse-treated total RNA was prepared from whole brains of mock- or prion-inoculated mice at 45, 100, 145 or 190 dpi and used for qPCR analyses. Serpin-*a3n* RNA levels are progressively elevated in brains of prion infected mice throughout pathogenesis and at 145dpi and 190 dpi are approximately 9-fold and 17-fold higher compared to mock inoculated controls. Murine measurements encompass a total of three biological replicates. All transcript levels were normalized to beta-actin and expressed relative to mock-inoculated mice or non-neurodegenerative controls. In the murine model of prion disease utilized, terminal signs are evident at approximately 200–210 dpi. Error bars represent standard deviations of three biological replicates. (C) Northern hybridization analysis of Serpin-*a3n* and Gfap in CNS areas of prion infected and uninfected mice at 90 dpi, 100 dpi and 140 dpi stages of pathogenesis. Equivalent loading of RNA is indicated by 18S rRNA levels. Abbreviations: Cb, cerebellum; Sc, spinal cord; Ob, olfactory bulb; Cx, cortex. Cystatin F differential expression in prion infected mice is first evident in the spinal cord between 90 dpi and 100 dpi (8-fold increase). By 140 dpi, Sepin*a3n* RNA levels in spinal cord, cerebellum and cortex are approximately 6-fold, 3-fold and 5-fold respectively. For comparison, by 140 dpi, Gfap RNA levels, an indicator of gliosis, in spinal cord, cerebellum and cortex are approximately 3-fold, 4-fold and 4-fold respectively. No change in expression levels of these transcripts in olfactory bulb at any of the stages of pathogenesis analyzed here. (D) DNAse-treated total RNA was prepared from occipital cortex obtained at autopsy from patients suffering from non-neurodegenerative diseases (Control), Alzheimer's disease (AD), or sporadic CJD (sCJD) patients. Left panel: Age distribution of the patients in each group at time of death. Right panel: box and whisker plots indicating upper- and lowermost values with mean and 75^th^ percentile for eight patients within each group. Values were tested for statistical significance using Student's *t* test (two samples, equal variance). Triplicate qPCR reactions were performed for each sample.

Both Northern hybridization and QPCR confirmed that whole-brain transcription of Serpin-*a3n* increased progressively during the course of prion infections. Significant overexpression was detected already between 120 and 130 dpi (Fig. 1A), and reached levels of up to 17-fold higher than controls by 190 dpi (Fig. 1B). Therefore, Serpin-*a3n* ranks among the most highly upregulated transcripts in prion-infected brains. For comparison, glial fibrillary acidic protein transcripts, a marker of reactive astrogliosis often used to quantitate brain damage, only reached levels of ≤8-fold higher than controls by late-stage of prion pathogenesis (Fig. 1A & B).

Having established that whole-brain Serpin-*a3n* RNA was markedly elevated upon prion infection, we investigated its regional expression in specific segments of the CNS. We found that Serpin-*a3n* RNA was upregulated 6-fold higher in spinal cord, 3-fold in cerebellum, and 5-fold in cortex of prion-infected mice by 140 dpi (Fig. 1C). For comparison, GFAP RNA was overexpressed 3-fold in spinal cord and 4-fold in cerebellum and cortex of the same mice (Fig. 1A & C). The very high upregulation found in the spinal cord may be related to the i.p. route of inoculation, which favors prion entry into the thoracic segments of the spinal cord.

The human homologue of Serpin-*a3n* is α_1_-anti-chymotrypsin (α_1_-ACT). We investigated α_1_-ACT mRNA in post-mortem occipital cortex of patients suffering from AD and sporadic CJD (sCJD). Brains of non-demented, age-matched patients were used for control (Fig 1D, left panel). Similarly to the observations reported above for prion-infected mice, α_1_-ACT RNA was significantly elevated in the CNS of sCJD patients (*p* = 0.019, Fig. 1D).

### Cystatin C and α_1_-ACT levels are unaltered in AD and sCJD patient plasma

Plasma cystatin C and α_1_-ACT were measured by sandwich ELISA and expressed either as raw concentration (ng/ml), or relative to total plasma protein (Table 1). For control, we assayed plasma from individuals with non-neurodegenerative diseases. Samples were from patients of similar age and gender distribution to the AD and sCJD groups, and were obtained at mid- and late-stage of disease. However, no alterations in the plasma concentration of either cystatin C or α_1_-ACT were evident in the sCJD and AD groups relative to controls.

**Table 1:** Plasma concentration of Cystatin C and α_1_-ACT in patients suffering from AD or sCJD.

	Control (n = 10)	AD (n = 10)	sCJD (n = 10)
**Patient age (years)**	70.90 (1.98)	77.40 (2.12)	69.00 (4.98)
**Total protein (g/L)**	65.79 (1.75)	66.16 (2.40)	64.31 (2.52)
**Cystatin C (µg/L)**	1310.26 (91.64)	1394.46 (59.00)	1381.25 (108.51)
**Cystatin C (ng/mg plasma protein)**	19.93 (1.23)	21.15 (0.71)	21.65 (1.68)
**α_1_-ACT (mg/L)**	978.42 (61.30)	931.08 (88.28)	1000.59 (96.48)
**α_1_-ACT (µg/mg plasma protein)**	14.80 (0.67)	14.15 (1.24)	15.86 (1.85)

Plasma levels of cystatin C and α_1_-ACT. Cystatin C and α_1_-ACT levels were measured in plasma of sCJD patients, as well as in age and gender-matched collectives of healthy persons and AD patients by ELISA. Numbers in parenthesis represent s.e.m.

### Cystatin C and α_1_-ACT in cerebrospinal fluid

We used sandwich ELISA to measure cystatin C and α_1_-ACT in the CSF of patients suffering from AD, sCJD and vCJD at mid- to late-clinical stage of disease. Values were expressed either as absolute concentrations or relative to total CSF protein (Table 2). The age and gender composition of sCJD patients, AD patients, and control group A (patients suffering from non-neurodegenerative diseases) was similar (p>0.05). Control group B represents patients of similar age and gender distribution to the vCJD collective, and was derived from patients initially suspected of having CJD but later determined to suffer from other diseases. Since vCJD tends to occur at a much younger age than sCJD, the average age of the two control groups is very different. Interestingly, the total CSF protein concentration and the concentrations of cystatin C were different between control groups A and control group B, suggesting that they may be modulated by an age-dependent process (Table 3).

**Table 2:** Cystatin C and α_1_-ACT in patient cerebrospinal fluid.

	Control A (n = 10)	AD (n = 10)	sCJD (n = 8)	Control B (n = 8)	vCJD (n = 10)
**Patient age (years)**	66.20 (4.36)	67.44 (3.17)	66.50 (3.91)	37.25 (4.25)	31.50 (2.44)
**Total protein (mg/L)**	871.58 (39.63)	771.94 (47.56)	917.14 (227.61)	500.87 (82.02)	452.99 (61.08)
**Cystatin C (µg/L)**	3875.00 (362.90)	3278.89 (345.50)	3868.75 (333.85)	4413.75 (642.03)	3113.00 (328.04)
**Cystatin C (ng/mg CSF protein)**	4471.19 (402.78)	4275.30 (406.21)	5275.04 (793.47)	9781.25 (1254.68)	8005.71 (1243.42)
**α_1_-ACT (mg/L)**	7.67 (0.33)	7.17 (0.45)	11.86 (3.05)	10.31 (2.86)	6.97 (0.35)
**α_1_-ACT (µg/mg CSF protein)**	8.90 (0.43)	9.30 (0.17)	12.98 (0.71)**	21.94 (3.85)	17.41 (1.84)

CSF levels of cystatin C and α1-ACT. Levels of cystatin C and α1-ACT were measured in patient CSF by ELISA and expressed as quantity per ml fluid or relative to total CSF protein. Values from sCJD patient CSF were compared to values obtained from CSF of both AD patients and healthy control groups of comparable age and gender distribution within groups (control group A). Since vCJD is typically prevalent in patients of a younger age, values for vCJD CSF were compared to those obtained from patients of comparable age (control group B). Numbers in parenthesis represent s.e.m.

**Table 3:** Cystatin C, α_1_-ACT and α_1_-microglobulin in the urine of patients.

	Control A (n = 10)	AD (n = 9)	sCJD (n = 29)	Control B (n = 10)	vCJD (n = 6)	Control C (n = 13)
**Patient age (years)**	73.50 (2.00)	80.20 (2.14)	67.52 (1.76)	23.50 (0.60)	28.67 (2.47)	52.69 (6.43)
**Cystatin C (µg/L)**	43.18 (3.46)	46.80 (8.00)	74.88 (9.87)	62.78 (18.92)	120.29 (29.79)	69.86 (18.74)
**Cystatin C (µg/mg albumin)**	6.21 (1.42)	5.67 (0.90)	4.18 (0.76)	7.00 (2.13)	15.00 (4.14)	7.52 (1.82)
**Cystatin C (µg/g Creatinine)**	40.74 (6.44)	40.59 (4.64)	91.18 (11.45)	79.13 (15.76)	80.28 (19.91)	70.70 (11.09)
**α_1_-ACT (µg/L)**	1008.68 (124.83)	913.01 (176.05)	16557.89 (3569.36)*	617.21 (26.11)	1431.98 (677.58)	2206.62 (849.88)
**α_1_-ACT (µg/mg albumin)**	131.21 (26.58)	111.70 (21.56)	560.68 (84.62)**	78.10 (15.21)	122.83 (22.26)	213.93 (47.94)*
**α_1_-ACT (µg/g Creatinine)**	888.73 (133.56)	778.05 (108.90)	20200.2 (4503.22) **	942.92 (84.48)	1959.31 (1534.83)	2125.57 (401.41) *
**α_1_-microglobulin (µg/g Creatinine)**	13.17 (6.45)	nd	53.86 (54.02)	5.83 (1.56)	37.33 (24.76)	nd
**α_1_-microglobulin (mg/g Creatinine)**	3.11 (1.44)	nd	4.40 (1.05)	0.44 (0.19)	3.23 (0.52)**	nd

Cystatin C and α1-ACT were measured in patient urine by ELISA and expressed as quantities per ml urine or relative to concentrations of albumin and creatinine. Values obtained from sCJD patient urine are directly comparable to values obtained from urine of AD patients and the healthy control group A consisting of patients of comparable age and gender distribution. Control group B represents a comparable control group for the vCJD measurements, consisting of patients initially suspected to have CJD but being later determined not to have CJD. Control group C consists of patients either pre-transplant with kidney disease, patients post-kidney transplant and patients with bladder carcinomas and nephritic inflammation. Numbers in parenthesis represent S.E.M. nd, not done.

**Figure 2:**
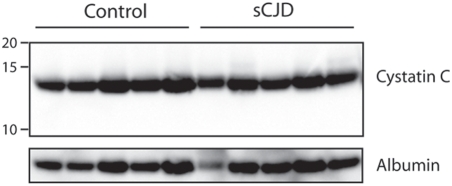
Western blot analysis of cystatin C in the CSF of patients suffering from sCJD. Equivalent amounts of CSF protein (10 µg) from late-stage sCJD patients and from appropriate age-matched non-sCJD patients were fractionated under non-reducing SDS-PAGE conditions and analyzed by Western blot using a polyclonal anti-human cystatin C antibody. No difference was found in CSF cystatin C levels of sCJD patients vs. controls.

In contrast to previous reports [15], [16], we found no elevated CSF levels of cystatin C in sCJD and vCJD patients (Table 2). Furthermore, Western blot analysis using immunoreagents derived from the same source as in one of these studies [16] showed levels of cystatin C in CSF of sCJD patients indistinguishable from non-CJD controls (Fig. 2). α_1_-ACT concentrations were then divided by total CSF protein concentration, and each patient collective was compared to the appropriate control group. After the latter normalization we found a moderate, yet significant, elevation of α_1_-ACT in CSF samples of sCJD patients (46% , *p*<0.001, Table 2).

### Urinary levels of cystatin C, α_1_-antichymotrypsin and α_1_-microglobulin

**Figure 3:**
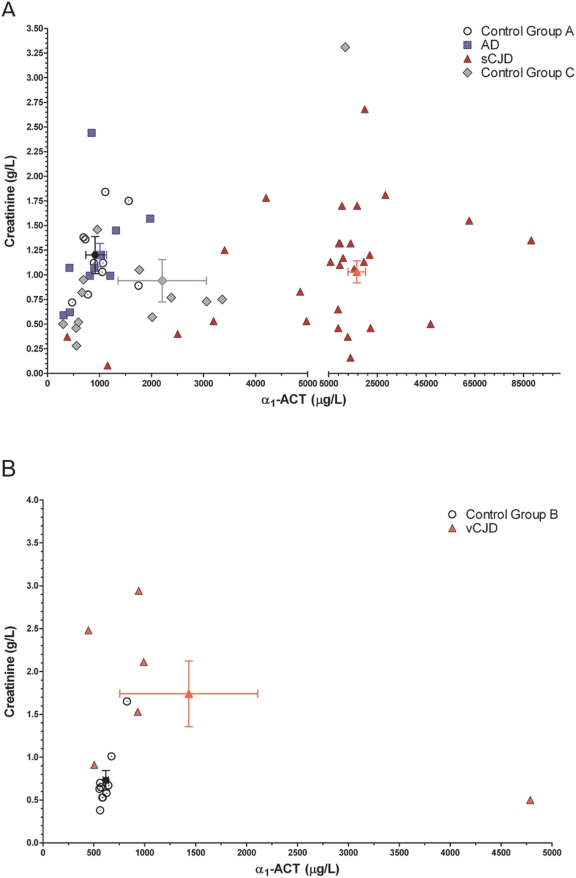
Urinary α_1_-ACT and creatinine levels. α_1_-ACT levels (µg/L) were plotted relative to creatinine levels (g/L) for (a) sCJD patients together with Control group A, AD, and Control group C patients and (b) vCJD patients together with respective Control group B patients. Error bars represent s.e.m.

The urinary concentration of cystatin C and α_1_-ACT in AD, sCJD and vCJD patients at mid-to-late stages of disease was determined by sandwich ELISA. Similarly to the CSF analysis reported above, urinary α_1_-ACT concentrations of sCJD patients were compared to those of AD patients and of control group A. We found dramatically elevated urinary α_1_-ACT in sCJD patients (Table 3, Figure 3). This was particularly evident when α_1_-ACT concentrations were normalized to albumin or to creatinine (*p*<0.001), but it reached significance even when raw concentrations were analyzed without any attempt at normalization (*p*<0.05).

We considered several artifactual scenarios which may have led to the above observations. Since both cystatin C and α_1_-ACT are present in urine at concentrations far below those observed in plasma (See Table 2), alterations may simply reflect general proteinuria resulting from compromised renal function. We addressed this in several ways. Firstly, prospectively recruited patients with known macro- or microhaematuria, or proteinuria, and retrospectively collected urine samples with albuminuria of ≥150 µg/ml, were excluded from further analysis. Secondly, we obtained urine from an additional set of patients (control group C) which predominantly included post-kidney-transplant patients following diabetic nephropathy or focal segmental glomerulosclerosis and reflux nephropathy. Thirdly, we investigated urine from patients presenting with bladder carcinoma, and with pyelonephritis with bacteriuria (Control group C). These groups also served to assess the specificity of the changes observed in the CJD groups relative to other non-neurological conditions.

Since many factors can affect urine concentration and, consequently, concentration of test substances, we normalized cystatin C and α_1_-ACT concentration against urinary creatinine. In the absence of renal dysfunction, the excretion rate of creatinine is relatively constant and has been used for adjusting urine concentration/dilution effects in measurement of urinary metabolites [28]. Normalization to creatinine therefore controls for both potential variations in urine concentration between individuals in spot-test urine samples and renal function. For these reasons we also expressed cystatin C and α_1_-ACT concentrations relative to both albumin and creatinine (Table 3). Urinary α_1_-ACT levels were evident in sCJD patients regardless of normalization to urinary albumin or creatinine. Additionally, we observed that neither age, sex or study centre sampling location were variables contributing to elevated urinary α_1_-ACT levels in sCJD patients (not shown).

**Figure 4:**
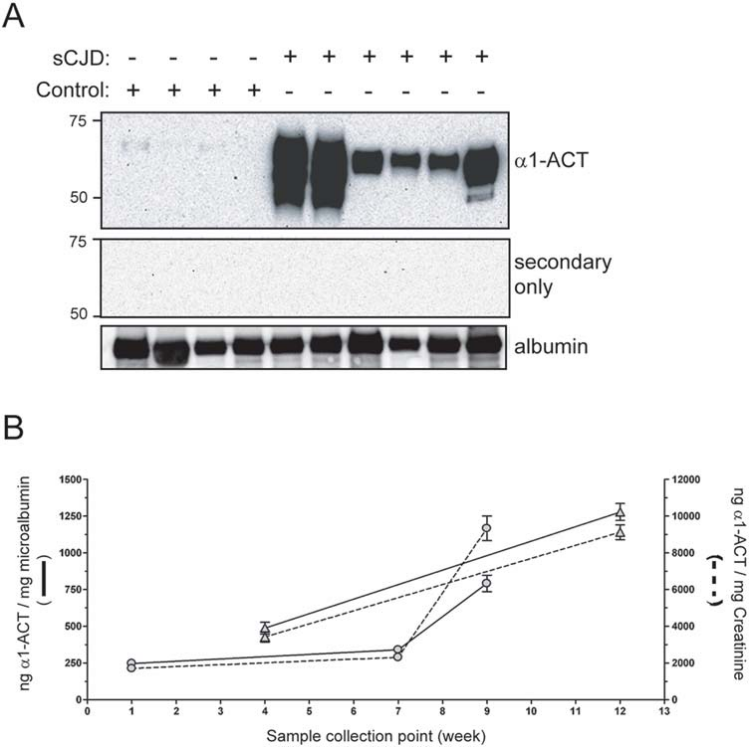
Urinary α_1_-ACT of sCJD patients. (A) Equivalent amounts (50 µg) of urinary protein from sCJD patients and appropriate age-matched controls were fractionated under non-reducing SDS-PAGE conditions and subsequently analyzed by Western blot using an anti-human α_1_-ACT monoclonal antibody. A duplicate blot incubated with HRP-labeled secondary antibody alone is shown. To verify equivalent protein loading, blots were stripped and re-probed with anti-albumin antibodies. Molecular weight marker positions are indicated. α_1_-ACT levels are significantly higher in sCJD patient urine, corroborating the data obtained by ELISA measurement. (B) ELISA measurement of α_1_-ACT in urine collected sequentially during the clinical phase of disease of two sCJD patients (represented as circles or triangles) indicates a progressive increase in α_1_-ACT levels normalized both for albumin and creatinine. Error bars represent the standard deviations in replicate measurements of each sample.

Since ELISA results can be confounded by crossreactive contaminants, we performed Western blotting analyses on selected urine samples. These confirmed dramatically elevated immunoreactive bands with the electrophoretic motility of α_1_-ACT in sCJD patients (Fig. 4A).

In contrast, we observed no statistically significant alteration in urinary cystatin C between sCJD or vCJD groups and respective controls, regardless of whether cystatin C concentration was expressed as raw, non-normalized data, or relative to either urinary albumin or creatinine (Table 3).

In addition, urinary α_1_-ACT was also significantly higher in sCJD patients compared to control group C when expressed either relative to albumin or creatinine (Table 3, *p*<0.001).

**Figure 5:**
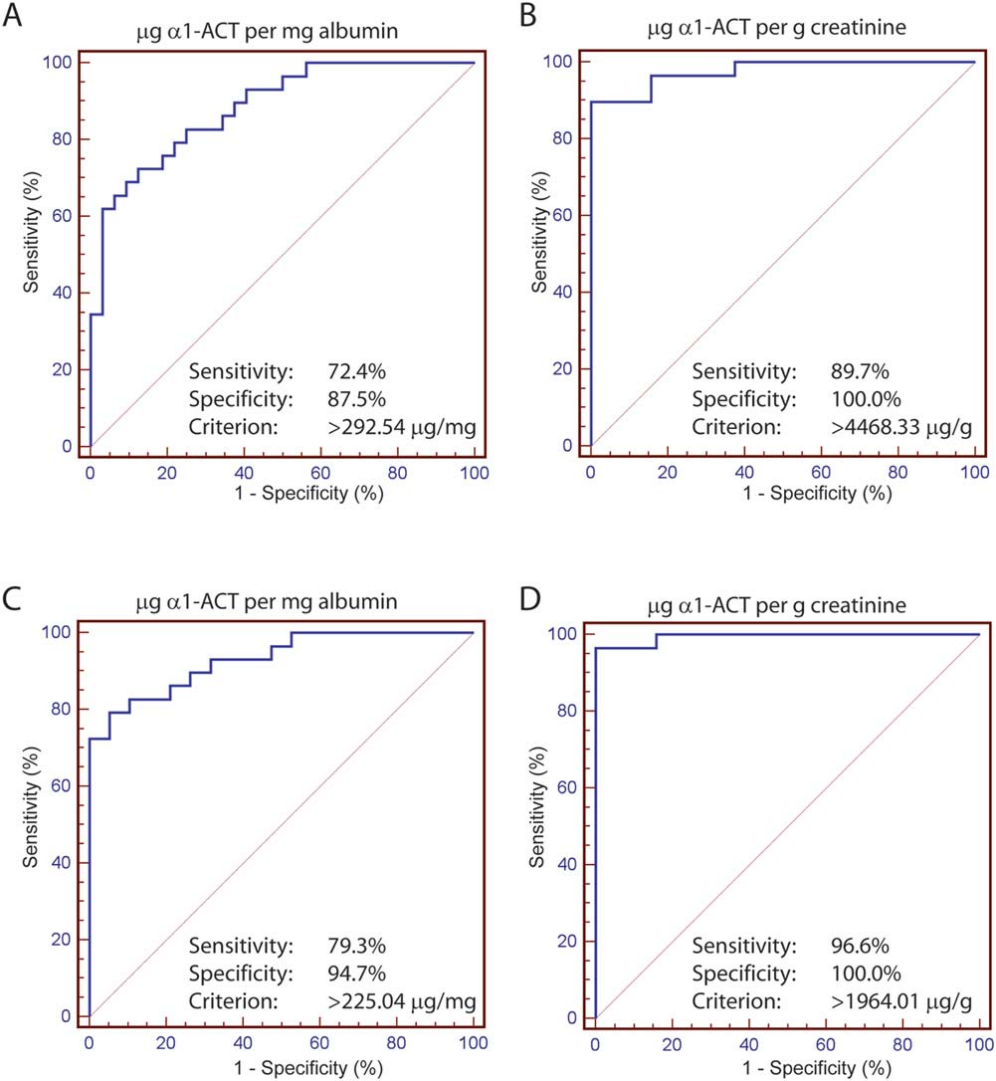
Receiver Operating Characteristic (ROC) Curve Analysis. ROC curves were calculated for µg α_1_-ACT/mg albumin (a & c) or µg α_1_-ACT/mg creatinine (b & d) for sCJD patients relative to either Control group A and AD patients (a & b) or Control group A, AD and Control C patients (b & d). Sensitivity, specificity, and threshold (criterion) for each are indicated.

When sCJD patients were compared to control groups A, C, and AD patients, the sensitivity and specificity of elevated urinary α_1_-ACT for sCJD were, respectively, 72.4%, 87.5% (µg/mg albumin) or 89.7%, 100% (µg/g creatinine) if Control group C is not included in the calculation, and 79.3%, 94.7% (µg/mg albumin) or 96.6%, 100% (µg/g creatinine) if Control group C is included in the calculation (Fig 5). In contrast to the data obtained for sCJD patients, we observed no significant alterations in urinary α_1_-ACT from vCJD patients referenced to control group B (Table 3, Figure 3).

Longitudinal measurements of two sCJD patients presented to the NRPE (one definite and one probable sCJD) revealed a progressive increase in urinary α_1_-ACT relative to both albumin and creatinine throughout the clinical course of disease (Fig. 4B). This finding suggests that urinary α_1_-ACT excretion may be useful for monitoring disease progression, (e.g. in therapeutical trials) as a responsive surrogate biomarker.

As a further indication of renal proximal tubule function, we measured urinary levels of α_1_-microglobulin in the sCJD and vCJD groups relative to respective control groups. The concentration of α_1_-microglobulin, normalized to creatinine or albumin, were not significantly altered in sCJD urine (Table 3), yet appeared to be higher in urine of vCJD patients than in appropriate controls (p<0.001). Normalization of values to both albumin and creatinine, and comparison to control group C, indicates that the observed increase in urinary α_1_-ACT concentrations in sCJD patients is independent of renal function. We also retrospectively assessed plasma creatinine and urea levels in 19 CJD patients presented to the NRPE. This analysis did not bring forward any evidence that gross renal impairment may represent a general feature of late-stage sCJD (Table 4).

**Table 4:** Selected NRPE sCJD patient plasma creatinine and urea levels.

NRPE Patient ID	sCJD status	Plasma Creatinine (52–114 µmol/L)	Plasma Urea (2.9–7.7 mmol/L)
217	Definite	58	3.6
222	Definite	113	nd
223	Probable	71	6.7
228	Definite	70	nd
233	Definite	81	5.5
234	Definite	124*	12*
238	Definite	92	nd
239	Definite	52	4
240	Probable	85	nd
246	Definite	95	10.3*
247	Definite	100	7.4
248	Definite	75	3.6
249	Definite	96	6.1
257	Probable	70	5.5
260	Definite	88	3.6
262	Definite	78	nd
268	Definite	72	nd
270	Probable	74	nd
271	Definite	61	4.5

Gross indication of renal function in sCJD patients was assessed by retrospective analysis of plasma creatinine and urea levels measured as part of the clinical assessment of sCJD patients. Normal reference ranges are indicated. Data obtained from patients during the clinical phase of disease and which have subsequently died and been confirmed post-mortem as definite sCJD are indicated. Those patients which are either still alive, or which have died but did not undergo post-mortem, are indicated as probable sCJD. Patients with plasma creatinine or urea levels out-with this expected range are indicated by an asterisk. Patients for which creatinine or urea measurements were not obtained are indicated (nd; not done)

### Urinary α_1_-ACT in field cases of animal prion diseases

**Figure 6:**
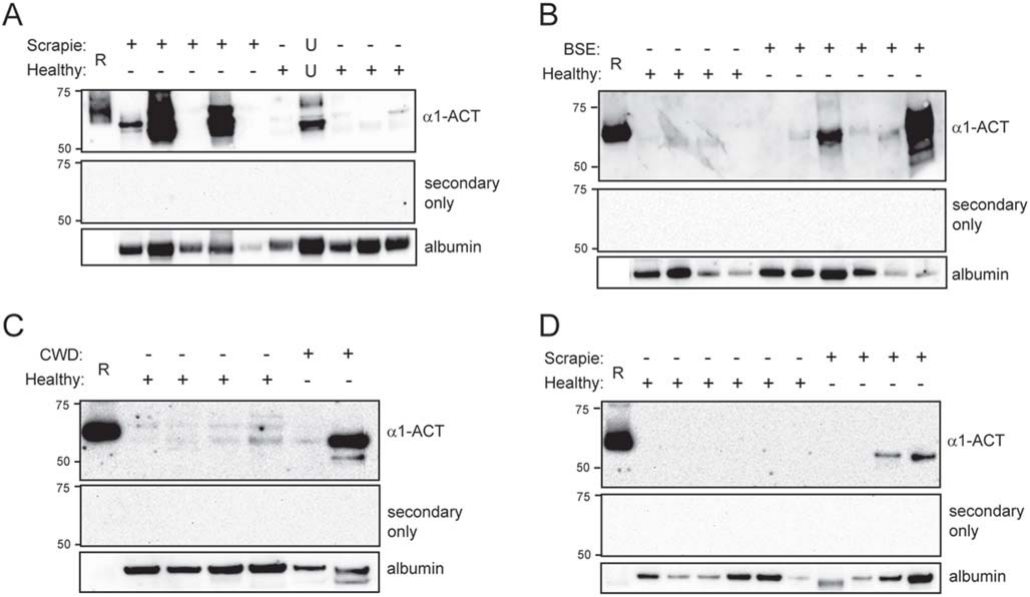
Western blot analysis of urinary α_1_-ACT from natural field cases of prion disease. Equivalent amounts of urinary protein from natural terminal field cases of (A) sheep scrapie (65 µg), (B) cattle BSE (30 µg), (C) deer CWD (90 µg) and (D) pre-mortem sheep scrapie (65 µg), together with urinary protein from appropriate healthy controls, were fractionated under reducing SDS-PAGE conditions and subsequently analyzed by Western blot using an anti-human α_1_-ACT monoclonal (A, B & D) or polyclonal (B) antibody. In each case a duplicate blot incubated with HRP-labeled secondary antibody alone was also prepared and is shown. To verify equivalent protein loading, blots were stripped and re-probed with an anti-human albumin antibody. Recombinant α_1_-ACT (30 ng) was loaded as positive control. Molecular weight marker positions are indicated. Panel A, lane 8 represents urine from a suspected scrapie case, the precise status of which is unclear (U) at present. Pre-terminal sheep scrapie urine (D) was analyzed from six individual healthy sheep and from one case at 10, 9, 2 & 1 months prior to being sacrificed at end-stage of disease (lanes 8–11 respectively).

We carried out Western blot analyses of urine from scrapie-sick sheep, cattle with confirmed Bovine Spongiform Encephalopathy (BSE) and deer with confirmed Chronic Wasting Disease (CWD), along with urine from healthy animals collected post-mortem (Fig. 6A–C). In addition, we analyzed by Western blot urine samples collected sequentially from one sheep that was pre-clinically infected with scrapie and went on to display clinical signs of disease. Scrapie was confirmed post-mortem in this sheep by histopathology and Western blot analysis of brain tissue (data not shown). Urine from the latter sheep was analyzed at 10, 9, 2 & 1 months prior to the development of terminal scrapie, together with urine from six sheep unaffected by scrapie (Fig. 6D). Urinary α_1_-ACT was increased 2 months before terminal disease, and remained sustained thereafter.

We also observed significantly elevated levels of urinary α_1_-ACT in two of six BSE-affected cattle (Fig. 6B) and in one of two CWD-affected deer (Fig. 6C). One animal from the sheep study shown in Fig. 6A was culled following appearance of scrapie-like symptoms. However, post-mortem biochemical and pathological confirmation was inconclusive, and this particular animal was classified as “atypical” scrapie. It is therefore unclear whether elevated urinary α_1_-ACT in this animal represents a false positive, or the identification of a scrapie case hitherto unqualified by other biochemical and histological techniques.

## Discussion

A global microarray screen of scrapie-infected mouse brains identified a limited number of genes undergoing transcriptional elevation well before the clinical onset of disease. One of these was Serpin-*a3n*, which encodes the serine proteinase inhibitor α_1_-antichymotrypsin (α_1_-ACT). Its transcription was drastically elevated not only in whole-brain homogenates of scrapie-infected mice, but also in occipital cortices of sCJD patients. These observations suggest that α_1_-ACT elevation occurs in prion infections of many species.

Serpin-*a3n* encodes a secretory leader peptide, and α_1_-ACT is present in body fluids. This raised the possibility that α_1_-ACT may represent a candidate biomarker of prion infection. Since many brain metabolites are found in the CSF, we first tested whether CSF concentrations of α_1_-ACT would predict prion infection. Indeed, sCJD patients exhibited a highly significant (*p*<0.001) increase of α_1_-ACT in CSF when normalized against total CSF protein and compared to appropriate age-matched controls.

α_1_-ACT is elevated in brains of AD patients and contributes to AD amyloid plaque deposits [17], [18], but the value of α_1_-ACT as a biomarker of AD is unclear. Some studies have reported elevated α_1_-ACT in plasma or CSF of AD patients [19], [20], [21], [22], whereas others found no changes [29]. In agreement with the latter study, we found no increased α_1_-ACT in plasma and CSF of AD patients in the present study.

We then sought to compare the diagnostic value of α_1_-ACT with that of cystatin C, which had been claimed to be a biomarker for CJD. We found that plasma and CSF levels of cystatin C did not discriminate between AD patients and age and gender-matched control patients, in agreement with previous findings [30]. Also, CSF measurements of cystatin C by sandwich ELISA in large collectives of sCJD, vCJD, and control samples was not discriminatory between these three groups. The latter finding is at striking variance with two studies of cystatin C in the CSF of small collectives of CJD patients (n = 8 and 6, respectively) [15], [16] reporting up to 30-fold increases of CSF cystatin C in sCJD patients.

Cystatin C undergoes proteolytic processing, and a 12.5 kDa cleavage product was reported to be elevated in the CSF of patients suffering from multiple sclerosis, even if the levels of full-length 13.4 kDa cystatin C were unaltered [31]. Therefore, antibodies differentially recognizing cystatin C and its degradation products may introduce a confounding factor. However, we were unable to demonstrate any differences in cystatin C levels in CSF of sCJD patients by Western blotting even when using the same antibody that had led to the claim of a 30-fold elevation of CSF cystatin C in sCJD patients [16]. We conclude that CSF cystatin C is unaltered in the collective of patients investigated here (n = 8) and therefore does not represent a biomarker of sCJD or vCJD.

We then studied the occurrence of immunoglobulins, α_1_-ACT, and cystatin C in urine samples. We were unable to confirm the previously reported presence of protease-resistant immunoglobulin light chains in the urine of prion-affected animals and humans [32], [33], [34]. Our use of anti-mouse IgG2a as secondary antibody rather than total IgG may account for this discrepancy. Also, cystatin C excretion was unaltered urine of AD, sCJD, and vCJD patients regardless of the normalization method used, and therefore not diagnostic of prion infection.

Surprisingly, urine samples from sCJD patients belonging to the UK, German and Swiss collectives displayed consistently elevated α_1_-ACT. Significance was attained independently of whether ELISA results were expressed as raw concentrations, or normalized against creatinine or albumin. ROC curve analysis confirms high specificity and sensitivity of the ability of urinary α_1_-ACT measurement to discriminate sCJD status. In a ROC curve the true positive rate (Sensitivity) is plotted in function of the false positive rate (100-Specificity) for different cut-off points. Each point on the ROC plot represents a sensitivity/specificity pair corresponding to a particular decision threshold. A test with perfect discrimination (no overlap in the two distributions) has a ROC plot that passes through the upper left corner (100% sensitivity, 100% specificity). Therefore the closer the ROC plot is to the upper left corner, the higher the overall accuracy of the test. The α_1_-ACT ELISA assay predicted prion infection with excellent sensitivity and specificity. The highest degree of sensitivity and specificity was observed for urinary α_1_-ACT normalised to creatinine. Vastly increased excretion of α_1_-ACT observed by ELISA-based measurement, this was also confirmed by Western blot analysis.

The enhanced excretion of α_1_-ACT in prion infections is surprising. In view of the unaltered plasma α_1_-ACT levels of sCJD patients, α_1_-ACT “spillover” from blood to urine is unlikely to underlie our observations. We therefore considered, and attempted to control for, possible artifactual effects. First, we considered that increased α_1_-ACT excretion may result from glomerular dysfunction. However, normalization of data to either urinary creatinine or albumin, which would be increased in many renal diseases, did not affect the results. Secondly, urinary cystatin C, a marker of glomerular filtration rate [35], was unaltered in sCJD patients, suggesting that increased α_1_-ACT excretion does not result simply from compromised renal function. Thirdly, elevated α_1_-antichymotrypsin levels in sCJD urine are unlikely to result from proximal tubule defects, since urinary α_1_-microglobulin levels were not significantly different in urine of sCJD patients relative to respective controls. Fourthly, our retrospective analyses of sCJD patient plasma creatinine and urea indicate that renal insufficiency is not a typical feature of late-stage sCJD patients (Table 4). Finally, urinary α_1_-ACT levels were significantly higher in sCJD than (1) in post-kidney transplant subjects following diabetic nephropathy, focal segmental glomerulosclerosis, and reflux nephropathy, (2) in patients with bladder carcinoma, and (3) patients with pyogenic nephritis leading to bacteriuria. These aggregated data argue against increased levels of α_1_-ACT in urine from sCJD patients simply resulting from renal disease, or from generally compromised health.

We considered that enhanced α_1_-ACT detection may result from sample collection or processing anomalies. We deem this unlikely for a number of reasons. Firstly, high urinary α_1_-ACT was diagnostic in sCJD patient collectives from each of three separate national reference centers. Secondly, no alterations of α_1_-ACT concentration in test urine were found when comparing voided vs. non-voided (P>0.05) urine, pre- vs. post-pellet urine (P>0.05), and urine samples collected in the morning or evening (P>0.05). Thirdly, the α_1_-ACT ELISA yielded similar results whether urines was stored immediately at −80°C or left at room temperature or 4°C overnight before testing (P>0.05). Therefore, none of the common sample collection and processing artifacts account for the reported results. Finally, elevated urinary α_1_-ACT was evident in a number of BSE-affected cattle, CWD-affected deer, and scrapie-affected sheep. In the latter, it was diagnostic at least 2 months prior to terminal disease.

Enhanced α_1_-ACT excretion may prove useful for correlating responsiveness to experimental therapeutic regimens and perhaps for biochemical discrimination of vCJD from sCJD without invasive biopsies. Finally, considering its excellent specificity and good sensitivity, one might consider exploring the usefulness of urinary α_1_-ACT determinations as a means of deferring blood units from asymptomatic donors who may be harboring preclinical prion infections.

## Materials and Methods

### Prion inoculations

Mice were maintained under specific pathogen-free conditions. Eight week-old inbred C57Bl/6 mice were inoculated intraperitoneally with 100 µl (6 log LD_50_ units) of the Rocky Mountain laboratory (RML) scrapie strain (passage 5 in CD1 mice, hence called RML5) prepared as a 10% (w/v) clarified brain homogenate containing 5% BSA. The titer of this standard RML5 inoculum was 8.9 log LD50 g^−1^ of brain tissue [36]. Age- and gender-matched control mice were inoculated intraperitoneally with 100 µl of a 10% (w/v) clarified brain homogenate from healthy CD1 mice. The incubation period of this model until onset of terminal disease was approximately 200–210 days post inoculation (dpi). Tissues from mice at 45, 100, 145 and 190 dpi stages of pathogenesis were used for real-time quantitative RT-PCR analyses. All manipulations were approved by the Animal Experimentation Committee of the Canton of Zürich.

### Tissue collection and RNA isolation

Human occipital cortex tissue was provided via the Swiss Reference Center for Prion Diseases (NRPE) and was collected at autopsy from a cohort of Swiss patients who died between 1996 and 2004. Patients with prion disease, Alzheimer's disease and patients without neurologic disorders were included. Tissues were processed according to established guidelines regarding safety and ethics and periods from death until post-mortem tissue collection were approximately 4–18 hours. All tissues were snap-frozen in liquid nitrogen prior to long-term storage at −80°C. Tissues from the RML murine model of prion pathogenesis were collected at various stages throughout pathogenesis. All murine tissues were immediately snap-frozen in liquid nitrogen prior to storage at −80°C until required. Total RNA was isolated using Trizol (Invitrogen AG, Switzerland) from eight patients of each control, AD and sCJD group.

### Preparation of labeled cRNA and microarray hybridization

All procedures for preparation of labelled cRNA probes and subsequent Genechip hybridisations were performed according to suggested Affymetrix guidelines (http://www.affymetrix.com). Total RNA isolated from whole brain using Trizol was subjected to a purification step (RNeasy mini kit, Qiagen, Switzerland). Quality of total RNA was assessed by agarose gel electrophoresis. Equal amounts of total RNA from 3 individual brains of mice at 145 dpi stage of prion pathogenesis were combined to act as template for generation of probe for one biological replicate. Thus, from nine mock-inoculated and nine prion inoculated mice, a total of three sets of labelled cRNA probes were synthesized representing three biological replicates. Double-stranded cDNA was synthesized using 20 µg total RNA as template (cDNA Synthesis kit, Cat.No.11917-010, Invitrogen, Switzerland) and primer 5′-GGCCAGTGAATTGTAATACGACTCACTATAGGGAGGCGG(dT)_24_-3′. Biotin-labeled cRNA was synthesized, using Enzo BioArray HighYield RNA transcript labeling Kit (T7) (Enzo Life Sciences, Germany), purified using the RNeasy mini kit and quantified by spectrophotometry. Quality was assessed by agarose gel electrophoresis. Typical yields were in the range of 120–150 µg labeled cRNA. 15 µg of labeled cRNA was fragmented in 40 mM Tris-acetate, 100 mM KOAc, 30 mM MgOAc, pH 8.1 at 95°C for 35 min. Following fragmentation, an aliquot of one representative of the replicates of each sample was hybridized to Affymetrix Test3 arrays to determine the quality of the probes, reflected by the 3′/5′ ratios, and to test all buffers using conditions outlined in the Affymetrix expression analysis manual. The samples were then hybridized to Affymetrix MOE430A and MOE430B chips. All hybridizations were carried out for 16 h at 45°C at 60 rpm. Chips were then washed and incubated with streptavidin-phycoerythrin according to the manufacturers instructions on the Affymetrix GeneChip Fluidics Station 450 with and scanned using the Affymetrix GS Scanner 2500 in conjunction with Affymetrix Microarray Suite 5.0 software.

### Microarray Data Analysis

Intensity values of all chips were normalized with dChip software (http://www.dchip.org) [37] and applying the model-based expression analysis algorithm (PM-only or PM/MM model) the expression values were calculated.

Global brain gene expression profiles from mice 145 days post-inoculation, with either mock inoculum or the RML prion strain, were compared applying a 1.3-fold change as the lower limit, lower 90% confidence boundary of fold change and *p* values of <0.05. Only those genes were considered which were flagged present in 100% of chips from cRNA probes derived from at least either mock or prion inoculated mice. Microarray datasets are available at www.ncbi.nlm.nih.gov/geo; accession number GSE7207.

### Northern Blot Analyses

Northern blot hybridisation experiments as previously described [38] using 15 µg total RNA. Random primed [α^32^P]dCTP probes were prepared from 20 ng of gel-purified DNA homologous to target transcripts using Rediprime II random priming kit (Amersham Biosciences, Switzerland) and purified with Nick Sephadex G50 columns (Amersham Biosciences, Switzerland). Prehybridization and hybridization was performed overnight at 42°C using Ultrahyb (Ambion, USA), containing 1×10^6^ cpm/ml radiolabelled probe and 100 µg/ml denatured herring sperm DNA (Catalysis AG, Switzerland) and 20 µg/ml denatured yeast tRNA (Sigma Fluka, Switzerland). Post-hybridization washes were performed according to Ultrahyb instructions. Hybridization signals were quantified using Fujifilm imaging plate technology (BAS-1800II, Fujifilm, Japan) and normalized for variations in RNA loading by subsequent probing with an excess of 18S rRNA cDNA probe [38].

### Probes and accession numbers

Murine cDNAs for derivation of probes for Serpin-*a3n* and Gfap were obtained from Open Biosystems (www.openbiosystems.com); accession numbers were BI145274 and AI836096 respectively.

### Quantitative Real-Time PCR (QPCR)

In all cases 5 µg of total RNA was used as template for first-strand cDNA synthesis. Prior to cDNA synthesis, residual genomic DNA was removed by the DNA free-kit (Ambion, USA). Total RNA was converted into cDNA using the bulk first strand cDNA synthesis kit and *Not* I-(dT)_18_ as primer (Amersham Biosciences Europe GmbH, Germany). Control reactions omitting reverse transcriptase were performed to validate successful removal of contaminating genomic DNA. Successful cDNA synthesis and lack of contamination with genomic DNA was tested by performing PCR (40 cycles) with primers specific for β-actin. All samples utilized for QPCR analysis were validated free of contaminating genomic DNA. Quantitative real-time PCR was performed using the SYBR Green PCR Master Mix (Qiagen AG, Switzerland) on an ABI PRISM 7700 Sequence Detection System (Applied Biosystems, Switzerland) using default cycling conditions. For murine samples, triplicate reactions of three biological replicates were utilized. In the case of human material, triplicate reactions were performed for each sample. Expression levels of serpin-*a3n*/α_1_-ACT were normalized to β-actin. Primer sequences were as follows: 5′- tatctgcctccacccaaaag-3′ & 5′- gccagatgtggacaaagtga-3′ (murine serpin-*a3n*), 5′-atggatgacgatatcgctg-3′ & 5′-atgaggtagtctgtcaggt-3′ (murine ActB), 5′-tctcccaggtggtccataag-3′ & 5′-ttactgagagccccactgct-3′ (human α_1_-ACT), 5′-ggacttcgagcaagagatgg-3′ & 5′-agcactgtgttggcgtacag-3′ (human ACTB).

### Enzyme-linked immunosorbent assays for α_1_-antichymotrypsin (α_1_-ACT)

Human α_1_-ACT was measured by triple antibody sandwich ELISA. Microtiter plate wells (Nunc F96 Maxisorb, Milan Analytica, Switzerland) were coated with 100 µl of 5.4 µg/ml affinity purified rabbit anti-human α_1_-ACT (Dako, Switzerland) in carbonate-bicarbonate buffer, pH 9.6. The plates were then sealed and incubated overnight at 4°C. After six washes with phosphate-buffered saline (PBS) containing 0.05% Tween-20 (PBST) the remaining binding sites in the wells were blocked by incubating with 100 µl of PBST containing 5% bovine serum albumin (BSA, Sigma) for 2 h at room temperature with shaking, followed by six washes with PBST. Thereafter, 100 µl of purified recombinant human α_1_-ACT (R&D Systems, UK), in several dilutions ranging from 0 to 200 ng/ml to generate a standard curve, and test samples were added. Standard curve and test samples were diluted into PBS sample buffer containing a final concentration of 0.05% Tween-20 and 1% BSA. Serum, CSF and urine were diluted 1∶250 000, 1∶3000 and 1∶300 respectively such that all samples were contained within the measurable range of the standard curve. Standard curve and test sample dilutions were prepared in triplicate. The plates were sealed and incubated at room temperature for 2 h at room temperature with shaking and washed six times with PBST. Then 100 µl of sheep anti-human α_1_-ACT (The Binding Site, UK), diluted to 31 µg/ml in PBST containing 1% BSA, was added to each of the wells and the plate sealed and incubated for 2 h at room temperature with shaking. Following a further six washes with PBST 100 µl of horseradish peroxidase (HRP) conjugated donkey anti-sheep IgG, diluted 1∶1000 in PBST containing 1% BSA, was added to each well and the plate sealed and incubated at room temperature for 2 h. Following a final six washes with PBST 100 µl of 3,3′,5,5′-teramethylbenzidine chromogenic substrate (TMB, Invitrogen, UK) was added to each well. Following colour development 100 µl of H_2_SO_4_ was added to each well to stop the reaction and the optical density measured at 450 nm or 405 nm in an ELISA micro-plate reader. Values were expressed as concentration per ml fluid (plasma, CSF & urine), relative to total protein (plasma & CSF) or relative to creatinine and albumin (urine). Inter- and intra-assay correlation of variance was 7.36% and 3.84% respectively.

### Enzyme-linked immunosorbent assays for cystatin C

Measurement of human cystatin C was performed using a commercial sandwich ELISA kit (Biovendor Laboratory Medicine Inc., Czech Republic) according to the manufacturer's instructions. Human cystatin C standard curve and test samples were diluted into PBS sample buffer containing a final concentration of 0.05% Tween-20 and 1% BSA. Serum, cerebrospinal fluid (CSF) and urine were diluted 1∶300, 1∶1000 and 1∶100 respectively such that all assay values were contained within the measurable range of the standard curve. Standard curve and test sample dilutions were prepared in triplicate. Optical density was measured at 450 nm or 405 nm in an ELISA micro-plate reader. Values were expressed as concentration per ml fluid (plasma, CSF & urine), relative to total protein (plasma & CSF) or relative to creatinine and albumin (urine).

### Albumin & α_1_-microglobulin

Measurements of human urinary albumin and α_1_-microglobulin were performed using a commercial competitive ELISA for albumin (Orgentec Diagnostika, Germany) and a sandwich ELISA format for α_1_-microglobulin (ImmunDiagnostik, Germany) according to the manufacturer's instructions. Optical density was measured at 450 nm in an ELISA micro-plate reader. Values were expressed as concentration per ml urine (albumin) or normalized to albumin or creatinine (α_1_-microglobulin).

### Urinary creatinine

Measurement of urinary creatinine was performed using a commercial kit (Cayman Europe, Estonia) based on the Jaffe alkaline picrate reaction according to the manufacturer's instructions. Urine samples were diluted 1∶15 in molecular biology grade water such that assay values were contained within the measurable range of the standard curve. Optical density was measured at 490 nm in an ELISA micro-plate reader. Values were expressed as concentration per ml urine.

### Western blots

Total protein concentration was determined by bicinchoninic acid (BCA) or microBCA (Perbio Science, Switzerland). Equal amounts (µg) of total protein were separated by electrophoresis through 12% SDS-PAGE gels and transferred to nitrocellulose membranes. Reducing conditions were used in all cases except for detection of human α_1_-ACT. The membranes were blocked in PBST containing 5% non-fat milk for 2 h at room temperature prior to incubating overnight at 4°C with primary antibody. Following six minute washes with PBST the membranes were incubated in PBST containing 5% non-fat milk and the appropriate secondary antibody for 1 h at room temperature. In the case of cattle urine, Top-block (GE Healthcare, UK) was used instead of 5% non-fat milk. As a positive control, purified recombinant human α_1_-ACT (R&D Systems, UK) was also included.

For detection of α_1_-ACT in human, sheep and deer urine, murine anti-human α_1_-ACT (R&D Systems, UK) was used at a concentration of 2 µg/ml followed by incubation with HRP-conjugated rabbit anti-mouse IgG_2a_ (Invitrogen, UK) at a dilution of 1∶5000. For detection of α_1_-ACT in cattle urine, rabbit anti-human α_1_-ACT (Dako, Switzerland) was used at a dilution of 1∶1000, followed by incubation with HRP-conjugated donkey anti-rabbit IgG (GE Healthcare, UK) at a dilution of 1∶1000. For detection of cystatin C in human CSF, samples were prepared under non-reducing conditions, electrophoresed and transferred to nitrocellulose membranes. After blocking as outlined above, membranes were incubated in PBST containing 5% non-fat milk and a 1∶1000 dilution of rabbit anti-human cystatin C (Upstate, UK), followed by washing with PBST and incubation with HRP-conjugated donkey anti-rabbit IgG (GE Healthcare, UK) at a dilution of 1∶1000. Following addition of ECL substrate (Perbio, Switzerland) signal was visualized using a VersaDoc model 4000 Imaging system (BioRad, Switzerland). Membranes were stripped (Western blotting stripping solution, Socochim, Switzerland) and re-probed with rabbit anti-human albumin (Abcam, UK), at a dilution of 1∶5000, followed by HRP-conjugated donkey anti-rabbit IgG (GE Healthcare, UK), at a dilution of 1∶1000, and subsequent ECL detection as outlined above.

### Body fluid samples of patients

Samples were obtained from patients who presented to the NRPE, the United Kingdom CJD Surveillance Unit (CJDSU), the German Reference Centre for Prion Diseases, the Clinic for Nephrology, the Institute for Clinical Pathology, and the Division of Psychiatric Research at University of Zürich. Collection of blood, CSF and urine for subsequent analysis was approved by the Swiss Federal Office of Health.

For control, we assembled two distinct groups of persons. Control group A consisted of individuals whose age and gender matched those of patients suffering from sCJD or AD, whereas the demographics of control group B matched patients suffering from variant CJD (vCJD). Specifically, control group B consisted of patients who initially presented to the UK CJDSU with suspected CJD, but were later determined to suffer from other ailments.

Urine and CSF samples were obtained from patients at varying clinical stages of CJD, from mid-clinical stage to terminal disease. In the case of terminal patients, body fluids were collected at post-mortem. Plasma was derived from patients ante mortem. From pre-mortem patients where sCJD was suspected, samples were included in the study only following post-mortem confirmation of sCJD.

In addition, we investigated a control group consisting of urine collected from patients post-kidney transplant following diabetic nephropathy, focal segmental glomerulosclerosis and reflux nephropathy conditions, from patients presenting with bladder carcinoma and bacteriuria, and from patients with cerebrovascular disease or encephalitis.

Urine from patients with known macro- or microhaematuria, or proteinuria, was excluded from further analysis. In retrospectively collected urine samples in which this was unknown, samples containing >150 µg/ml albumin were excluded from the study. Within approximately 18 h of collection, urine was centrifuged briefly, aliquoted and stored at −80°C until analysis. Blood was collected in tubes containing citrate to a final concentration of 0.5% and, within 12 h of collection, centrifuged a 2 280 *g* for 4 minutes at room temperature. The plasma layer was aliquoted and stored at −80°C until required. Cerebrospinal fluid was collected, aliquoted and stored at −80°C. Informed consent was obtained in all relevant cases.

### Field Animals

Urine samples were obtained from sheep scrapie cases both pre-terminal (without the use of diuretic drugs or catheter) or at post-mortem via bladder puncture. Urine from deer CWD and cattle BSE cases (and controls) were obtained at post-mortem by bladder puncture. Pre-terminal scrapie cases consisted of six healthy Sarda sheep held in the Sassari region of Italy (*PRNP* genotypes: ARR/ARQ, ARQ/VRQ, ARR/ARR, ARQ/ARQ) and one natural scrapie positive animal (ARQ/ARQ), from which urine was collected pre-clinical and throughout the clinical phase of disease. Urine from terminal sheep was derived from UK sheep of the Suffolk breed and consisted of males and females displaying no clinical signs of scrapie (ARR/ARR and ARQ/ARQ *PRNP* genotypes) and natural scrapie positive males and females (ARQ/ARQ *PRNP* genotypes).

### Statistical tests

Data was analyzed for statistical significance using two-tailed unpaired Mann-Whitney *t*-test with 95% confidence interval. Significance of *p*<0.05, and *p*<0.001 is indicated by * and ** respectively. Receiver operator characteristic (ROC) curves were calculated and plotted using the software MedCalc.

## Supporting Information

Table S1 (0.08 MB XLS)
